# Noninvasive Assessment of the Fractional Flow Reserve with the CT FFRc 1D Method: Final Results of a Pilot Study

**DOI:** 10.5334/gh.837

**Published:** 2021-01-04

**Authors:** Daria Gognieva, Yulia Mitina, Timur Gamilov, Roman Pryamonosov, Yuriy Vasilevskii, Sergey Simakov, Fuyou Liang, Sergey Ternovoy, Natalya Serova, Ekaterina Tebenkova, Valentin Sinitsyn, Ekaterina Pershina, Sergey Abugov, Gaik Mardanian, Narek Zakarian, Vardan Kirakosian, Vladimir Betelin, Dmitry Shchekochikhin, Abram Syrkin, Philippe Kopylov

**Affiliations:** 1 Department of Cardiology, Functional and Ultrasound Diagnostics of N.V. Sklifosovsky Institute for Clinical Medicine, I.M. Sechenov First Moscow State Medical University (Sechenov University), Moscow, RU; 2 Department of Physiology, School of Biomedical Sciences; University of Melbourne, Melbourne, AU; 3 Laboratory of Mathematical Modeling in Biomedicine, I.M. Sechenov First Moscow State Medical University (Sechenov University), Moscow, RU; 4 Shanghai Jiao Tong University, Shanghai, CN; 5 Department of Radiology and Radiotherapy of N.V. Sklifosovsky Institute for Clinical Medicine, I.M. Sechenov First Moscow State Medical University (Sechenov University), Moscow, RU; 6 Department of Radiation Diagnostics and Therapy, Faculty of Fundamental Medicine, M.V. Lomonosov Moscow State University, Moscow, RU; 7 N.I. Pirogov City Clinical Hospital № 1, Moscow, RU; 8 Department of endovascular diagnostic and treatment, Russian Medical Academy of Continuous Professional Education, Moscow, RU; 9 B.V. Petrovsky Russian Research Center of Surgery, Moscow, RU; 10 Clinical hospital № 1, Moscow, RU; 11 Scientific Research Institute of Systematic Research, RAS, Moscow, RU

**Keywords:** Noninvasive Assessment of Fractional Flow Reserve, Coronary Artery Disease, Coronary Computed Tomography Angiography

## Abstract

**Background:**

Until recently, Russia did not utilize noninvasive fractional flow reserve (FFR) assessment. We developed an automated algorithm for noninvasive assessment of FFR based on a one-dimensional (1D) mathematical modeling.

**Objective:**

The research aims to evaluate the diagnostic accuracy of this algorithm.

**Methods:**

The study enrolled 80 patients: 16 of them underwent 64-slice computed tomography – included retrospectively, 64 – prospectively, with a 640-slice CT scan. Specialists processed CT images and evaluated noninvasive FFR. Ischemia was confirmed if FFR < 0.80 and disproved if FFR ≥ 0.80. The prospective group of patients was hospitalized for invasive FFR assessment as a reference standard. If ischemic, patients underwent stent implantation. In the retrospective group, patients already had invasive FFR values.

Statistical analysis was performed using GraphPad Prism 8. We compared two methods using a Bland–Altman plot and per-vessel ROC curve analysis. Considering the abnormality of distribution by the Kolmogorov-Smirnov test, we have used Spearman’s rank correlation coefficient.

**Results:**

During data processing, three patients of the retrospective and 46 patients of the prospective group were excluded. The sensitivity of our method was 66.67% (95% CI: 46.71–82.03); the specificity was 78.95% (95% CI: 56.67–91.49), p = 0.0052, in the per-vessel analysis. In per-patient analysis, the sensitivity was 69.57% (95% CI: 49.13–84.40); the specificity was 87.50% (95% CI: 52.91–99.36), p = 0.0109. The area under the ROC curve in the per-vessel analysis was 77.52% (95% CI: 66.97–88.08), p < 0.0001.

**Conclusion:**

The obtained indices of sensitivity, specificity, PPV, and NPV are, in general, comparable to those in other studies. Moreover, the noninvasive values of FFR yielded a high correlation coefficient with the invasive values. However, the AUC was not high enough, 77.52 (95% CI: 66.97–88.08), p < 0.0001. The discrepancy is probably attributed to the initial data heterogeneity and low statistical power.

## Introduction

Noninvasive assessment of the fractional flow reserve (FFR) is an emerging diagnostic tool highly informative for the anatomical structure of coronary arteries, localization of stenotic lesions, and functional significance of each lesion. This approach is particularly promising in patients with borderline lesions, and in those cases, when exercise stress test cannot be performed or its results are noninformative.

The technique is based on constructing a patient-specific computational model of coronary blood flow using data obtained via routine computed tomography (CT) with further numerical calculation of FFR value based on information acquired from this model.

The first method (FFRCT), and the only approved by the Food and Drug Administration (FDA), was developed by the HeartFlow company (USA). It has been demonstrated high diagnostic accuracy and a high degree of correlation with the invasive FFR measurement in major studies [1,2,3,4]. A computational procedure implies manual construction of a 3-dimensional (3D) mathematical model of coronary blood flow resulting in lengthy data processing (24 hours, dropped to 1–4 hours), which requires high-performance computing resources. Data analysis costs may reach 1 500 US dollars per patient [5].

Another RUO technique (cFFR) was developed by the Siemens Healthineers (Germany). The method implies the calculation of FFR through both 3-dimensional and 1-dimensional (1D) modeling. Unlike the HeartFlow, cFFR works on a standard desktop personal computer and takes less time (from 30 minutes to two hours) due to 1D modeling and simplification of a calculation mechanism [6]. This approach has also demonstrated its efficiency though the studies enrolled a smaller cohort of patients (5).

Until recently, Russia did not utilize noninvasive FFR assessment. In 2015 we developed an automated algorithm for noninvasive assessment of FFR based on 1D mathematical modeling—CT FFRc 1D [7,8]. This technique currently passes the validation phase in clinical settings.

The current article describes the final results of a pilot study aimed at determining the diagnostic efficiency of CT FFRc 1D and the degree of correlation between the noninvasive indices and the invasive ones used as a reference standard.

## Methods

We conducted a pilot single-center interventional study consisted of two main phases. The local ethics committee of I.M. Sechenov First Moscow State Medical University approved the study, approval reference number—10–17. The study was conducted in accordance with the provisions of the Declaration of Helsinki (1995). All patients gave written informed consent prior to the inclusion in the study. The data underlying this article cannot be shared publicly due to the decision of the local ethics committee. The data will be shared on reasonable request to the corresponding author. Detailed description of inclusion and exclusion criteria available on clinicaltrials.gov, NCT03797118.

### First phase

The first phase was retrospective. It included evaluation of computed tomography coronary angiography (CTCA) data in patients of the original prospective study by Pershina E.S. et al. (2018) [9]. This study comprised CTCA data of 16 patients (13 males and three females; mean age: 47.8 ± 2.3 years) with stenoses of 45% to 75% in arteries more than 2 mm in diameter. We had a minimum set of input data, which comprised sex, age and weight of the patient, information on the presence or absence of angina symptoms, and their severity. Briefly, all patients enrolled in the original study underwent CTCA on a 64-slice CT scanner (Discovery 850, the USA) with a minimum slice thickness of 0.5 mm per one rotation of an x-ray tube (0.275 sec). The non-enhanced (native) and arterial phases were performed with prospective ECG gating. An iodine-based radiocontrast agent (Iopamidol, 370 mg/mL, Sanochemia Pharmazeutika AG, Austria) at a dose of 1 mL/kg body weight and 100 mL of normal saline were bolus-administered sequentially via a peripheral venous catheter (size: 18 and 20 G, depending on a patient’s weight) with an automatic syringe at a flow rate of 5 mL/sec. All patients had Agatston scores less than 400 Hounsfield units (HU) according to the primary data. Within two weeks after CTCA, all patients were hospitalized in interventional radiology departments to undergo invasive FFR measurement as a reference standard. FFR values <0.80 were considered significant. Consequently, for the retrospective group of patients, we had CT scans and invasive FFR data.

### Second phase

The prospective phase was carried out at the University Clinical Hospital No. 1’s Clinic of Cardiology of the I.M. Sechenov First Moscow State Medical University.

The second phase implied prospective inclusion of a sample of 64 patients met the criteria as mentioned earlier. They underwent standard protocol CT scanning on an Aquilion ONE 640-slice scanner (Toshiba, Japan) with a minimum slice thickness of 0.5 mm per one rotation of an x-ray tube (0.275 sec). CTCA was conducted in native and arterial phases. The native phase was performed with prospective ECG gating to calculate calcium score, while the arterial phase—using retrospective ECG gating. An iodine-based radiocontrast agent (Iopamidol, 370 mg/mL, Sanochemia Pharmazeutika AG, Austria) at a dose of 1 mL/kg body weight and 100 mL of normal saline were bolus-administered sequentially via a peripheral venous catheter (size: 18 and 20 G, depending on a patient’s weight) with an automatic syringe at a flow rate of 4.5 mL/sec. The arterial phase started automatically when the radiodensity in the descending aorta reached 220 HU.

The mean effective radiation dose was 10–15 mSv both in the retrospective and prospective groups. Sublingual nitroglycerin at a dose of 0.5 mg was administered five minutes before scanning to patients of both groups who had no evidence of considerable hypotension (< 110/70 mm Hg), for a sufficient level of vasodilation. As a reference standard, patients in the prospective group underwent invasive FFR measurement. The time between CTCA and hospitalization in the interventional radiology department didn’t exceed two months.

### Invasive FFR measurements

For invasive FFR measurement, we used a Volcano s5 console and PrimeWire (Volcano Corporation, USA) intravascular guidewires (0.014”) with intracoronary pressure transducers. A sensor-tipped guidewire was connected to an interface (RadiAnalyzer™ Xpress Measurement System [St. Jude Medical Inc., USA] or ComboMap® Pressure and Flow System [Volcano Corporation, USA]). A diagnostic or a guiding catheter (size 6 F) was placed at the ostium of the coronary artery followed by intracoronary injection of 250 μg of nitroglycerin for coronary vasodilation. After a pressure transducer was set to nil automatically, and inserted in vessels. The pressure transducer was set immediately adjacent to the guiding catheter outlet, after which aortic, and transducer pressures were equalized. The pressure transducer was positioned no less than two cm distal to the targeted stenosis. Maximum hyperemia was induced with intracoronary papaverine (20 mg for the left coronary artery (LCA) and 12 mg for the right coronary artery (RCA)). After that FFR was registered.

If functionally significant stenosis (FFR <0.80) was detected, the patient underwent drug-eluting coronary stent implantation.

Ischemia was confirmed if there was at least one single stenosis of a large coronary artery for which FFR was less than 0.80 and disproved if none of the stenoses had FFR less than 0.80.

### Withdrawal

**Figure 1:**
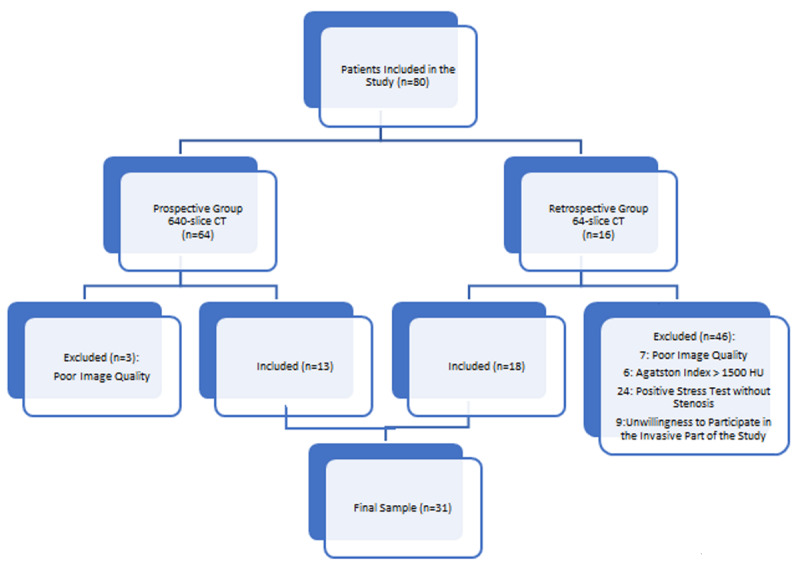
The Clinical Trial Outline.

Three patients from the retrospective group were withdrawn from the study due to poor CT image quality (Figure 1).

Among the prospective group, 46 patients were withdrawn. In essence, six patients with Agatston scores greater than 1 500 HU, 24 patients with positive exercise stress test who, according to CTCA, had no evidence of atherosclerotic lesions, seven patients for whom it was impossible to analyze data due to the poor-quality CT scans and nine patients who refused to participate in the invasive phase of the study. Thus, the prospective group included 18 subjects (Figure 1).

Data of patients (n = 31) in both groups were transferred to the Laboratory of Mathematical Modelling in Medicine (Sechenov University) for further processing, mathematical model building, and FFR computation utilizing the CT FFRc 1D method. Characteristics of the enrolled patients are presented in Table 1A, 1B and 1C.

**Table 1:** **A.** Characteristics of patients included in the retrospective phase of the study (n = 13); **B.** Characteristics of patients included in the prospective phase of the study (n = 18); **C.** General characteristics of all patients (n = 31). Mean ± standard deviation or frequency (%).

Parameter	A. Retrospective group (n = 13)	B. Prospective group (n = 18)	C. Joint patient group (n = 31)

Age, years	61.07 ± 9.70	65.44 ± 2.14	63.61 ± 1.65
Men, n (%)	9 (69)	12 (66.67)	22 (70.97)
Height, sm	170.00 ± 2.14	171.33 ± 2.33	170.74 ± 1.52
Weight, kg	86.69 ± 2.64	77.89 ± 2.91	81.58 ± 2.05
BMI, kg/m^2^	30.02 ± 0.83	26.64 ± 1.02	28.06 ± 0.71
Smoking, n (%)	–	5 (27.78)	–
Type 2 DiabetesMellitus, n (%)	–	1 (5.56)	–
Arterial hypertension*, n (%)	–	18 (100)	–
Angina clinic, n (%)	13 (100)	16 (88.89)	29 (93.55)
A history of MI, n (%)	1 (7.69)	3 (16.67)	4 (12,9)
A history of PCI, n (%)	0	2 (11.11)	2 (6.45)
LV EF, %	–	62.78 ± 1.80	–
Serum creatinine, μmol/l	–	83.57 ± 7.36	–
GFR (ml/min/1.73m^2^)^1^	–	82.44 ± 4.52	–
SBP^2^, mm Hg	135.00 ± 3.00	132.22 ± 2.17	133.39 ± 1.68
DBP^3^, mm Hg	86.15 ± 1.34	82.50 ± 1.33	84.03 ± 0.95
HR^4^, bpm	64.92 ± 0.68	65.39 ± 1.56	65.19 ± 0.90

^1^ Glomerular filtration rate (GFR) was calculated using the Modification of Diet in Renal Disease (MDRD) formula. ^*^ Arterial hypertension was diagnosed if arterial blood pressure ≥ 140/90 mm Hg was detected in at least two outpatient measurements. ^2^ Systolic blood pressure measured at the time of CTCA scan. ^3^ Diastolic blood pressure measured at the time of CTCA scan. ^4^ Heart rate measured at the time of CTCA scan. BMI – body mass index; MI – myocardial infarction; PCI – percutaneous coronary intervention; LV EF – left ventricular ejection fraction; GFR – glomerular filtration rate; SBP – systolic blood pressure; DBP –diastolic blood pressure; HR – heart rate.

### Data processing

Numerical calculation of FFR in the Laboratory of Mathematical Modelling consisted of the following stages:

1. Computer processing of CT data: 3D reconstruction of coronary arteries; averaged 1D reconstruction of coronary arteries using segmentation algorithms. CT images were processed in the following order:
  – preprocessing: when it was necessary, a few initial CT images with narrowed visibility scope were deleted and the lung vessels were shadowed using mathematical morphology;
  – segmentation of aorta, a search for ostium points and segmentation of coronary arteries: 3D modeling of the aorta and coronary arteries;
  – artery skeletonization: the vessel centerlines were extracted from the 3D model;
  – artery graph construction: the artery graph was built containing information on the vessel topology, diameter and length; sites of virtual FFR computation were marked with individual graph edges with appropriate modified diameters.
2. Personalization of coronary blood flow model: setting model parameters (vascular elastic modulus, arterial blood pressure, heart rate, degree of occlusion in the area of stenosis) using medical history and patient’s pertinence to certain statistical groups (age, alcohol consumption, tobacco use, body mass index).
3. Mathematical modeling of coronary hemodynamic parameters via a 1D dynamic vasculature hemodynamic model. Calculation of mean linear velocity and pressure in all coronary vessels that were reconstructed in the segmentation stage.
4. The FFR coefficient was calculated using data obtained from mathematical modeling.

All calculations were blinded; the invasive FFR data were not available to the specialists of the Laboratory. Afterward, the obtained results were compared to the already available values of the invasive FFR measurement.

### Statistical analysis

Statistical analysis was performed using GraphPad Prism 8. Continuous variables are presented as mean ± standard deviation, ordinal variables as median with interquartile range in parentheses. We considered p values <0.05 as statistically significant. Normality of data distribution was assessed using the Kolmogorov-Smirnov test with Lilliefors correction.

As a reference standard, we used an FFR value of <0.80. We applied the same threshold to evaluate sensitivity, specificity, positive predictive value (PPV), and negative predictive value (NPV).

Having calculations based on the retrospective data that were acquired via the 64-slice scanner (13 patients/16 vessels) performed first, on the prospective data acquired via the 640-slice scanner (18 patients/28 vessels) performed second and total calculations for both groups (31 patients/44 vessels) performed last, we evaluated per-patient and per-vessel sensitivity, specificity, PPV and NPV.

We used a Bland–Altman plot and per-vessel ROC curve analysis for two methods comparison. Considering the abnormality of distribution, we have used Spearman’s rank correlation coefficient to assess the degree of correlation between our model and the reference standard.

## Results

### Retrospective group

In the per-vessel analysis of the retrospective data obtained with a 64-slice scanner, the sensitivity was 100% (95% CI: 72.25–100); the specificity was 33.33% (95% CI: 59.23–70), p = 0.1250; the PPV was 71.43% (95% CI: 45.35–88.28); the NPV was 100% (95% CI: 17.77–100).

In the per-patient analysis, the sensitivity was 88.89% (95% CI: 56.50–99.43); the specificity was 25% (95% CI: 0.0128–69.94), p = 0.9999; the PPV was 72.73% (95% CI: 43.44–90.25); the NPV was 50% (95% CI: 0.02565–97.44).

In both cases, the obtained values did not meet statistical validity criteria.

**Figure 2:**
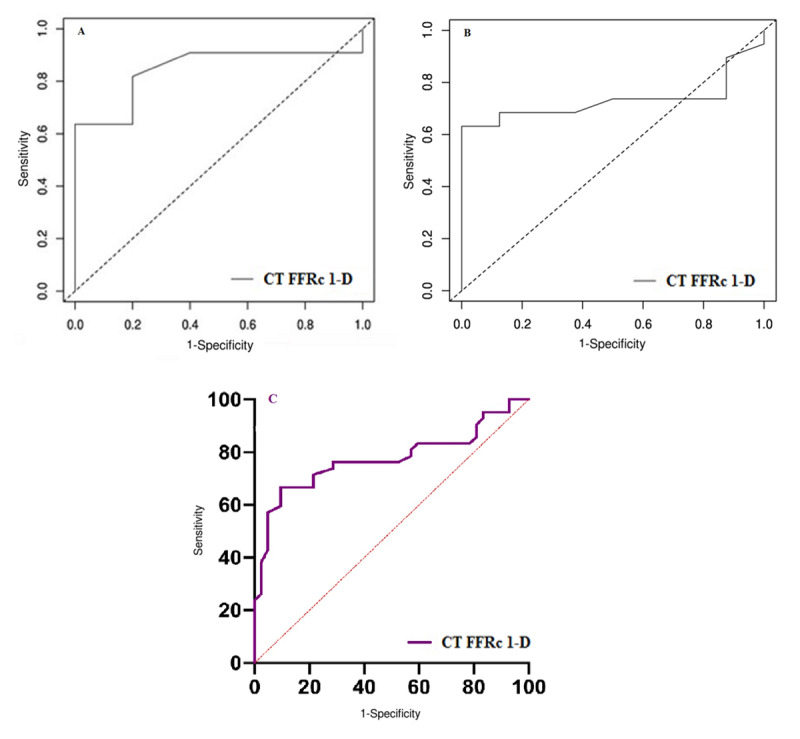
ROC curve analysis (per-vessel). **A.** For the retrospective group (n = 16) data, p < 0.001. **B.** For the prospective group (n = 28) data, p = 0.019. **C.** For the overall group (n = 44) data, p < 0.0001.

The area under the ROC curve in the per-vessel analysis was 84.54% (63.93–100), p < 0.001 (Figure 2A).

**Figure 3:**
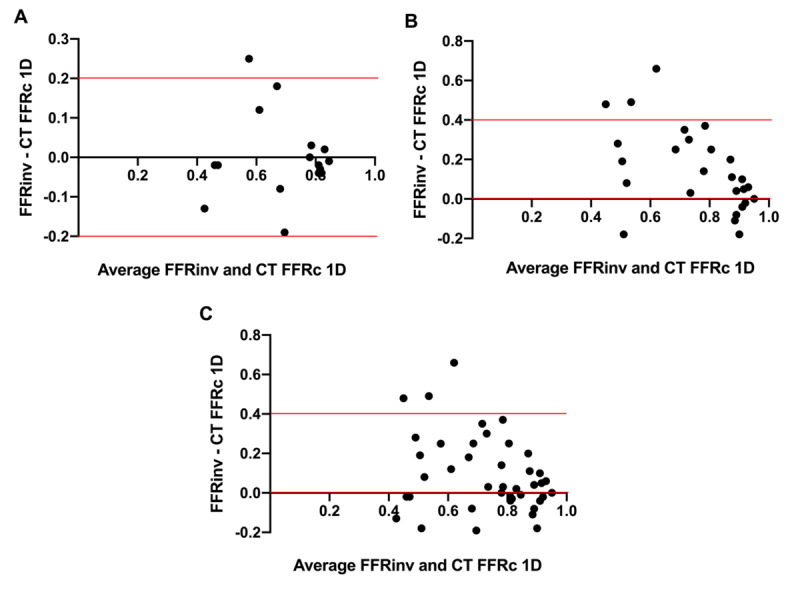
Bland–Altman analysis. **A.** Bland–Altman plot for the retrospective group (n = 16). **B.** Bland–Altman plot for the prospective group (n = 28). **C.** Bland–Altman plot for the overall group (n = 44).

According to the Bland–Altman plot, the mean difference of measurements in the retrospective group was 0.001250±0.108100 (Figure 3A).

### Prospective group

In the per-vessel analysis of the prospective data obtained with the 640-slice scanner, the sensitivity was 42.86% (95% CI: 21.38–67.41); the specificity was 100% (95% CI: 77.19–100), p = 0.0159; the PPV was 100% (95% CI: 60.97–100); the NPV was 61.9% (95% CI: 40.88–79.25).

In the per-patient analysis, the sensitivity was 50% (95% CI: 25.38–74.62); the specificity was 100% (95% CI: 60.97–100), p = 0.0537; the PPV was 100% (95% CI: 60.97–100); the NPV was 50% (95% CI: 25.38–74.62).

The area under the ROC curve in the per-vessel analysis was 73.02% (53.74–92.31), p = 0.019 (Figure 2B).

According to the Bland–Altman plot, the mean difference of measurements in the prospective group was 0.1415±0.2091 (Figure 3B).

### Overall group

In the per-vessel analysis of the overall data (n = 31), the sensitivity was 66.67% (95% CI: 46.71–82.03); the specificity was 78.95% (95% CI: 56.67–91.49), p = 0.0052; the PPV was 80% (95% CI: 58.40–91.93); the NPV was 65.22% (95% CI: 44.89–81.19).

In the per-patient analysis, the sensitivity was 69.57% (95% CI: 49.13–84.40); the specificity was 87.50% (95% CI: 52.91–99.36), p = 0.0109; the PPV was 94.12% (95% CI: 73.02–99.70); the NPV was 50% (95% CI: 26.80–73.20).

The area under the ROC curve in the per-vessel analysis was 77.52% (95% CI: 66.97–88.08), p < 0.0001(Figure 2C).

According to the Bland–Altman plot, the mean difference of measurements in the overall group was 0.09238±0.1908 (Figure 3C).

Spearman’s rank correlation coefficient between the virtual FFR values and the invasive ones was 0.5334 (95% CI: 0.2653–0.7249), p < 0.0003.

### Average time of CT FFRc 1D computation

The average time of CT FFRc 1D computation was 16 minutes per patient, with the average of 13 minutes for 64-slice CT and of 25 minutes for 640-slice CT. The difference exists due to the more complex and detailed vasculature visualization when derived from 640-slice CT. This estimation did not include two patients with 640-slice CT and one patient with 64-slice CT, for whom the data were processed manually due to the ingression of veins in the segmentation area. All calculations were performed on a personal computer with a 2.0 GHz CPU and 6.0 GB of RAM.

## Discussion

The obtained indices are, in general, comparable to those in other studies, Table 2. Though, the area under the ROC curve was quite low for the total group of patients, although, for the prospective and retrospective groups, it was high enough. The discrepancy is probably attributed to the initial data heterogeneity and low statistical power. More clinical studies with larger patient cohorts should be done to evaluate the fitness of this method.

**Table 2:** Comparison of the diagnostic efficiency for the CT FFRc 1D method with over non-invasive technics of the fractional flow reserve assessment described in the literature.

Study	DISCOVER–FLOW [1]	DeFACTO [2]	NXT [3]	Renker et al. [13]	Coenen et al. [14]	Ko et al. [15]	Kruk et al. [16]	Yang et al. [17]	CT FFRc 1D Method

**Year**	2011	2013	2013	2014	2015	2016	2016	2016	2019
**Patients number (n)**	103	252	254	53	106	42	90	72	31
**Vessels number (n)**	159	407	484	67	189	78	96	138	44
**Software**	HeartFlow v1.1	HeartFlow v1.1	HeartFlow v1.3	Siemens v1.4	Siemens v1.4	Toshiba Medical	Siemens v1.4	Siemens v1.4	CT FFRc 1D
**Sensitivity per vessels**	0.88 (0.77–0.95)	0.80 (0.73–0.86)	0.84 (0.75–0.89)	0.85 (0.62–0.97)	0.88 (0.78–0.91)	0.78 (0.51–92.6)	75.6	87 (75–94)	66.67 (47–82)
**Specificity per vessels**	0.82 (0.73–0.89)	0.61 (0.54–0.67)	0.86 (0.82–0.89)	0.85 (0.72–0.94)	0.65 (0.55–0.74)	0.87 (0.71–0.95)	72.3	77 (66–85)	78.95 (57–91)
**Sensitivity per patients**	0.93 (0.82–0.98)	0.90 (0.83–0.95)	0.86 (0.77–0.92)	0.94 (0.70–0.99)	N/A	N/A	75.6	N/A	69.57 (49–84)
**Specificity per patients**	0.82 (0.68–0.91)	0.54 (0.45–0.63)	0.79 (0.72–0.84)	0.84 (0.68–0.94)	N/A	N/A	71.4	N/A	88 (53–99)
**PPV per vessels**	0.74 (0.62–0.84)	0.56 (0.49–0.62)	0.61 (0.53–0.69)	0.71 (0.49–0.87)	0.66 (0.55–0.74)	0.74 (0.49–0.90)	67.4	71 (58–81)	80 (58–92)
**NPV per vessels**	0.92 (0.85–0.97)	0.84 (0.78–0.89)	0.95 (0.93–0.97)	0.93 (0.81–0.98)	0.88 (0.79–0.94)	0.89 (0.74–0.96)	80.0	90 (80–96)	65.22 (45–81)
**AUC per patients**	0.92	0.81	0.90	0.91	N/A	N/A	N/A	N/A	N/A
**AUC per per vessels**	0.90	N/A	0.93	0.92	0.83	0.88	0.835	0.893	66.25 (47.82–84.67)
**Accuracy**	84.3 (77.7–90.0)	–	86 (83–89)		74.6 (68,4–80,8)	83.9	74.0	81 (74–88)	–
**Correlation coefficient Pearson’s/Spearman’s^1^**	0.678	0.63	0.82	0.66	0.59	0.57	0.67^1^	0.671	0.6591^1^

PPV – positive predictive value; NPV – negative predictive value; N/A– not available.

Our research showed that, in the vast majority of cases, the automated algorithm could process data derived from 640-slice CT without manual refinement. However, due to the limited number of such diagnostic units even in Moscow, we have separated a retrospective group of patients, with 64-slice CT scanner-derived CTCA.

When processing the data, we have encountered specific difficulties. The first one was the impossibility of performing artery segmentation for the areas not filled with a dye agent.

The second was that the dye agent when entering veins, added those areas in segmentation. Graph edges associated with veins were manually deleted before FFR computation. Although the images obtained through the 640-slice scan are more informative compared to the 64-slice scan, the most crucial was the contrast-enhanced phase, as shown in Melikian N. et al. work [10]. Also, as already mentioned above, we lacked information on several hemodynamic parameters for a few patients, which lowered the precision of the estimates.

Two patients of the retrospective group had crucial differences between the estimated and the invasive FFR values that could affect treatment strategy. Three more patients had considerable differences between the values that did not affect the treatment strategy.

We would like to emphasize specifically the following case. Patient (CT FFRc 1D = 0.58; invasive FFR = 0.76) had significant stenosis of 80% with a hypothetically complex 3D geometry that hardly reproduced with a 1D model. A 1D model is ill-suited for the characterization of blood flow when significant stenosis has a complex geometry. Therefore, for the low values of FFR, significant deviations between the noninvasive and invasive FFR are frequently observed. This disadvantage is not crucial, because even demonstrating lower accuracy, CT FFRc 1D remains within the threshold of FFR < 0.6.

In the prospective group, we observe deviations affecting the treatment strategy in a patient with a previous Q-wave myocardial infarction (MI).

**Figure 4:**
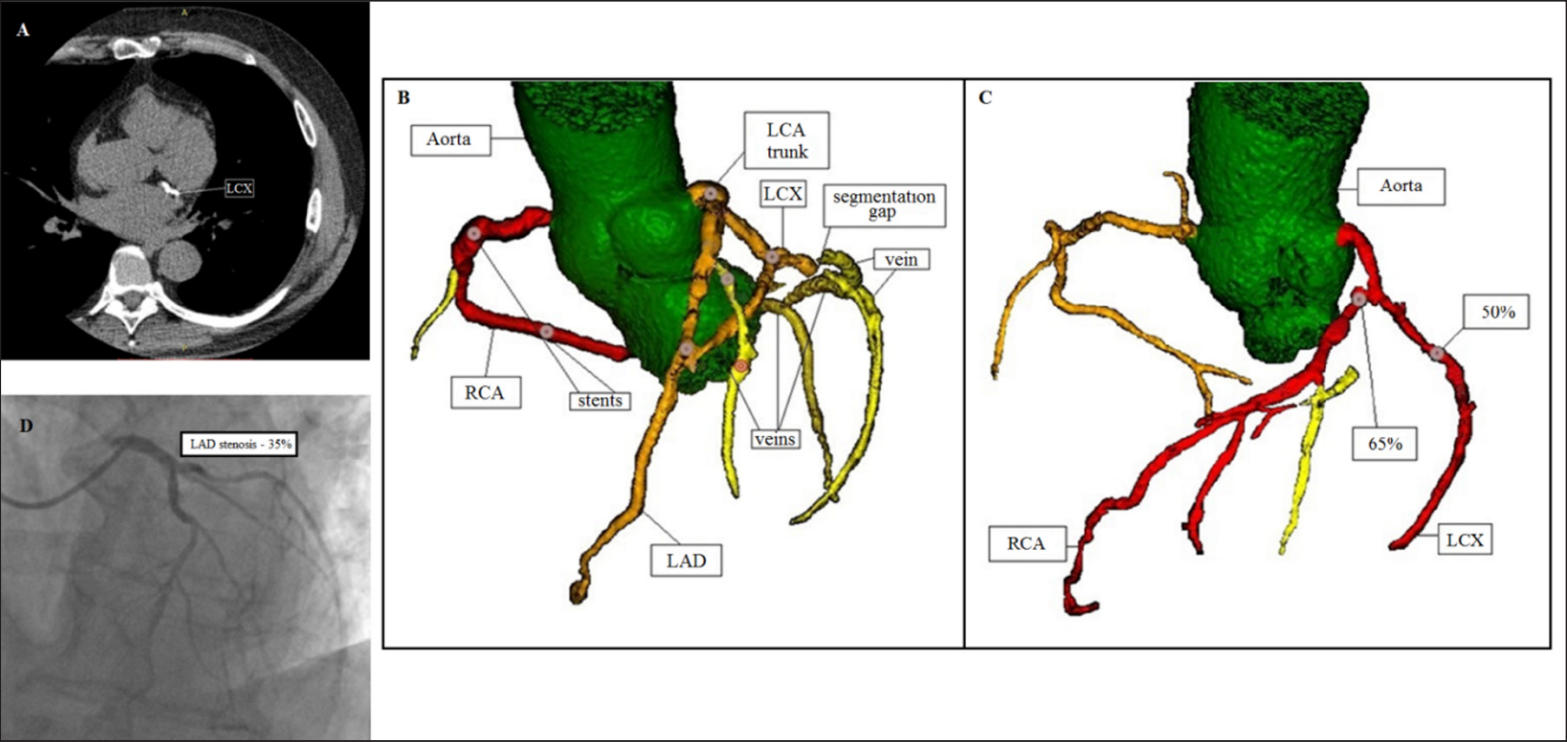
**A.** Native-phase CT images in DICOM format. Considerable calcification of the left circumflex artery (LCX). **B.** 3D reconstruction of coronary vessels. Segmented veins and the gap in segmentation of the left circumflex artery are visible (RCA – right coronary artery; LCA – left coronary artery; LAD – left anterior descending artery; LCX – left circumflex artery). **C.** 3D reconstruction of coronary vessels. CTCA showed 65% stenosis in the proximal segment of the left anterior descending artery (LAD – left anterior descending artery; LCX – left circumflex artery). **D.** Invasive coronary angiography. Stenosis in the proximal segment of the left anterior descending artery up to 35% (LAD – left anterior descending artery).

Patient had invasive FFR: left anterior descending artery (LAD) – 0.56, left coronary artery (LCA) trunk – 0.81, left circumflex artery (LCX) – 0.63; noninvasive FFR: LAD – 0.41; LCA trunk – 0.88; LCX – 0.81. In this case, the FFR deviation can be attributed to several factors. First, significant calcification (Agatston score = 1 401 HU) (Figure 4A), reduced left ventricular ejection fraction (38%) and, as a result, poor contrast enhancement caused gaps in segmentation. Moreover, contrast-enhanced vein areas were segmented. The lost fragment of the LCX had to be constructed manually in the graph construction phase; veins required additional data processing also (Figure 4B). Second, according to the medical records, the patient had a Q-wave myocardial infarction (MI) of the inferior wall. CTCA performed in 2018 showed a subendocardial filling defect in the apex of the heart and local apical thinning to 0.4–0.5 cm.

The diagnostic accuracy of the noninvasive FFR is lower in patients with prior Q-wave MI and further myocardial scarring apparently due to the lower volume-to-mass ratio, as compared with patients with stable coronary artery disease. Moreover, assumptions regarding the pattern of microvascular vasodilation responses that are used in blood flow modeling may be inaccurate or even false in patients with prior ST-elevation MI [11].

Though results obtained for the LCA trunk and LAD did not cross the threshold, the divergence was significant. A plaque located in the LCA trunk had evidence of instability (‘soft plaque’), i.e., was not calcified, so calcination degree could not affect the analysis. Keep in mind that invasive FFR is measured distal to the stenosis. LAD/LCX lesions located beneath the LCA trunk can affect results of invasive measurement, which also depend on the mass of myocardium perfused by this artery segment. At the same time, the noninvasive FFR is calculated in the most narrowed section of an artery without considering downstream stenoses.

A similar situation occurs when there are several sequential stenoses. The invasive FFR is measured when a sensor-tipped guidewire is gradually moved from the distal to the proximal segments under maximum hyperemia. Because each upstream stenosis will affect the pattern of hyperemic blood flow across the downstream lesion (distal stenoses have a more significant impact on FFR measurement than proximal stenoses), individual FFR assessment for each lesion is not applied in clinical practice, where the cumulative FFR is used instead.

Noninvasive FFR assessment implies a fundamentally different approach, wherein only separate stenoses are considered. A study by Simakov S. S. et al. demonstrated a case of noninvasive FFR assessment with a 1D model in a patient who had two sequential stenoses. The authors concluded that the formula for calculating FFR should be substantially modified to assess the functional significance of each of these stenoses [7,12].

## Conclusions

The obtained indices of sensitivity, specificity, PPV, and NPV are, in general, comparable to those in other studies. Moreover, the noninvasive values of FFR yielded a high correlation coefficient with the invasive values. However, the area under the ROC curve was not high enough, 77.52 (95% CI: 66.97– 88.08), p < 0.0001. The discrepancy is probably attributed to the initial data heterogeneity and low statistical power. More clinical studies with larger patient cohorts should be done to evaluate the fitness of this method.
